# Intensity-Dependent Effects of Acute Exercise on Executive Function

**DOI:** 10.1155/2019/8608317

**Published:** 2019-06-04

**Authors:** Aylin Mehren, Cecilia Diaz Luque, Mirko Brandes, Alexandra P. Lam, Christiane M. Thiel, Alexandra Philipsen, Jale Özyurt

**Affiliations:** ^1^Biological Psychology Lab, Department of Psychology, School of Medicine and Health Sciences, Carl von Ossietzky Universität Oldenburg, Oldenburg, Germany; ^2^Psychiatry and Psychotherapy, School of Medicine and Health Sciences, University Hospital Karl-Jaspers-Klinik, Carl von Ossietzky Universität Oldenburg, Oldenburg, Germany; ^3^Leibniz Institute for Prevention Research and Epidemiology-BIPS GmbH, Department of Prevention and Evaluation, Unit Applied Health Intervention Research, Bremen, Germany; ^4^Department of Psychiatry and Psychotherapy, University of Bonn, Bonn, Germany; ^5^Research Center Neurosensory Science, Carl von Ossietzky Universität Oldenburg, Oldenburg, Germany; ^6^Cluster of Excellence “Hearing4all”, Carl von Ossietzky Universität Oldenburg, Oldenburg, Germany

## Abstract

Numerous studies suggest beneficial effects of aerobic exercise at moderate intensity on cognition, while the effects of high-intensity exercise are less clear. This study investigated the acute effects of exercise at moderate and high intensities on executive functions in healthy adults, including functional MRI to examine the underlying neural mechanisms. Furthermore, the association between exercise effects and cardiorespiratory fitness was examined. 64 participants performed in two executive function tasks (flanker and Go/No-go tasks), while functional MR images were collected, following two conditions: in the exercise condition, they cycled on an ergometer at either moderate or high intensity (each *n* = 32); in the control condition, they watched a movie. Differences in behavioral performance and brain activation between the two conditions were compared between groups. Further, correlations between cardiorespiratory fitness and exercise effects on neural and behavioral correlates of executive performance were calculated. Moderate exercise compared to high-intensity exercise was associated with a tendency towards improved behavioral performance (sensitivity index *d*′) in the Go/No-go task and increased brain activation during hit trials in areas related to executive function, attention, and motor processes (insula, superior frontal gyrus, precentral gyrus, and supplementary motor area). Exercise at high intensity was associated with decreased brain activation in those areas and no changes in behavioral performance. Exercise had no effect on brain activation in the flanker task, but an explorative analysis revealed that reaction times improved after high-intensity exercise. Higher cardiorespiratory fitness was correlated with increased brain activation after moderate exercise and decreased brain activation after high-intensity exercise. These data show that exercise at moderate vs. high intensity has different effects on executive task performance and related brain activation changes as measured by fMRI and that cardiorespiratory fitness might be a moderating factor of acute exercise effects. Thus, our results may contribute to further clarify the neurophysiological mechanisms underlying the beneficial effects of exercise on cognition.

## 1. Introduction

A growing body of literature suggests a positive effect of a single session of physical exercise (acute exercise) on cognitive functions. Overall, small to medium effect sizes are proposed, which depend on several moderating factors like the type of cognitive function assessed, exercise characteristics, and cardiorespiratory fitness of the participants [1–4].

It is generally agreed that performance on complex tasks (i.e., executive functioning) is more likely to be affected by acute exercise than performance on more simple tasks (i.e., simple recall or reaction time) [3]. Authors have argued that the prefrontal cortex, which is of high relevance for executive functioning, might be particularly sensitive to neurophysiological changes induced by exercise. The latter might include increases in central arousal, increased release of neurotransmitters like dopamine, norepinephrine, and serotonin [5–7], and increases in brain-derived neurotrophic factor (BDNF) [8–10]. Furthermore, increases in cerebral blood flow (CBF) due to exercise have been proposed [11–13], while it is not clear yet if these changes are specific to prefrontal brain regions or rather global. Some authors argue that exercise elevates the proportion of CBF in prefrontal brain regions in comparison to other areas, which could account for enhanced executive functioning [4]. Probably related to the time course of neurophysiological changes, the timing of cognitive task administration seems to play a crucial role. A meta-analysis by Chang et al. [2] suggested that tasks administered 11-20 min after exercise cessation would evoke the greatest positive effects. However, it is not clear yet how long physiological and cognitive changes last.

Besides the type of cognitive function assessed and timing of task administration, characteristics regarding exercise like the type (aerobic vs. anaerobic), duration, and intensity possibly act to moderate the effects of exercise on cognition [2, 3]. To date, no standardized exercise protocols have been established. Concerning exercise intensity, several authors proposed an inverted-U relationship between exercise workload and changes in cognitive performance, suggesting that acute exercise at moderate intensity induces more beneficial effects than exercise at low or high intensity [14, 15]. This inverted-U model is based on the assumption that neurophysiological changes like increases in arousal and catecholamine levels only correspond to improved cognition up to an optimal point, following which further increases will lead to neural noise and deteriorate performance [16, 17].

Empirical studies and meta-analytical investigations have indeed shown that acute moderate exercise has an overall positive impact on cognitive functions, although results show a high variability and depend on the factors discussed above such as type and timing of the cognitive task. For acute high-intensity exercise, results are highly inconsistent, showing positive, negative, or no effects at all [3]. Moreover, very few studies have investigated the neural mechanisms underlying cognitive performance changes in response to either moderate- or high-intensity exercise. Two fMRI studies have found increased activation of parietal and hippocampal regions [18] and increased as well as decreased activation in frontal regions [19] during a working memory task after aerobic exercise at moderate intensity. MacIntosh et al. [20] observed decreased activation in the left parietal operculum during a Go/No-go task after moderate exercise compared to preexercise. A recent study by Chang et al. [21] found decreased prefrontal cortex oxygenation as measured by fNIRS during a Stroop task after high-intensity resistance exercise but not after high-intensity aerobic exercise. In addition, Kelly et al. [22] reported increased resting state brain activation in the prefrontal cortex and the substantia nigra in patients with Parkinson's disease after acute high-intensity exercise consisting of both resistance exercises and aerobic elements. Taken together, the results of the few neuroimaging studies available are highly heterogeneous, and fMRI studies comparing the acute effects of moderate- vs. high-intensity exercise are missing.

Another possible moderator that has received increasing attention is cardiorespiratory fitness. Higher levels of fitness have been associated with enhancements in the brain structure, brain function, and cognitive performance [11, 23–26]. fMRI evidence has established a relationship between higher fitness levels and increased recruitment of brain attentional networks during executive function tasks, with greater task-related activity in the prefrontal and parietal cortices and reduced activity in the anterior cingulate cortex [27, 28]. Furthermore, fitness might act as a moderator of acute exercise effects on cognition and also interact with exercise intensity, as trained individuals are used to higher workloads and perceive physical stress induced by exercise differently. Therefore, fitness might influence neuroendocrine responses to exercise and thereby contribute to cognitive effects. Hüttermann and Memmert [29] found that the inverted-U function only applied for nonathletes when measuring their attentional performance during exercise at different workload intensities, while expert athletes maintained performance during physical exercise regardless of exercise intensity. On the other hand, in a meta-analysis by Ludyga et al. [30], effects of moderate aerobic exercise on executive control were found to be not different between participants with different levels of fitness. Evidence from studies including neuroelectric measures suggests that exercise-induced improvements in allocation of brain resources depend on the fitness level of the participants [31, 32]. However, it has not been investigated yet how fitness is associated with exercise-related changes in brain activation as measured by fMRI.

The aim of the present fMRI study was to investigate intensity-dependent effects of an acute bout of exercise on executive functioning in healthy adults. Participants performed in a flanker and a Go/No-go task following a 30 min exercise protocol that involved ergometric cycling at either moderate or high intensity and following a control condition (watching a movie). We hypothesized that exercise at moderate intensity would lead to greater improvements in executive task performance and related changes in brain activation patterns than exercise at high intensity. Furthermore, we expected an association between cardiorespiratory fitness and exercise effects.

## 2. Material and Methods

### 2.1. Participants

Adult participants were recruited via announcements on the website of the University of Oldenburg and a local internet-based advertising portal. Inclusion criteria were (i) right-handedness, (ii) no use of psychotropic drugs, (iii) no MRI contraindications, (iv) no health conditions which could interfere with cycling tasks, and (v) absence of neurological and psychiatric disorders. Neurological and psychiatric disorders were assessed using the German versions of the Structured Clinical Interview for DSM-IV (SCID-I) [33], the SCID-II screening questionnaire for personality disorders [34], and the Beck Depression Inventory (BDI-II) [35]. To further evaluate health conditions relevant to physical activity, an electrocardiogram and a health questionnaire (German version of the Physical Activity Readiness Questionnaire) [36] were administered before participation.

The current study is part of a larger project on exercise effects on patients with attention deficit hyperactivity disorder and healthy controls [37]. For the purpose of this study, 64 healthy participants were assigned to either a moderate- or a high-intensity exercise group (each *n* = 32) based on age- and gender-matching. Note that one of the 32 participants in the high-intensity exercise group had to be excluded from further analyses due to drug abuse, which was not obvious prior to data acquisition. Demographic characteristics of all participants included in the analyses are summarized in Table 1. The study was conducted in accordance with the Declaration of Helsinki [38], and all procedures were approved by the ethics committee of the University of Oldenburg. All participants gave written informed consent prior to study participation.

### 2.2. Experimental Tasks

The experimental tasks were projected onto a screen and presented to the participants in the scanner through a mirror on the head coil using Cogent 2000 v125 (http://www.vislab.ucl.ac.uk/cogent.php) and Matlab R2015b (The MathWorks Inc.). The distance from the eyes of the participants to the screen was 50 cm. Stimuli were white and displayed on a black background. Participants used an MR-compatible keypad (NAtA Technologies, Coquitlam, Canada) to respond with their right hand.

#### 2.2.1. Flanker Task

To measure interference control and selective attention, we implemented an arrow version of the Eriksen flanker task [39]. Each stimulus consisted of five arrows arranged in a row (one target at the center and two flankers on each side). Three different trial types were presented: in congruent trials, the flankers and the target pointed into the same direction; in incongruent trials, the flankers pointed into the opposite direction as the target; and in neutral trials, flankers were lines without arrowheads. The target pointed to the left or to the right with equal probability and was smaller than the flankers to amplify the interference effect. Flanker task stimuli were presented using an event-related design. Each trial started with the presentation of a fixation cross for 1.5 sec, followed by a stimulus for 0.5 sec. The participants were instructed to focus on the fixation cross and, as soon as the stimulus appears, to indicate the direction of the target by pressing one of two keys (index finger for left, middle finger for right). They were told to respond as fast and accurate as possible. The duration of the complete task was 10 min, consisting of 300 trials (100 per trial type). Both the sequence of the trial types and the direction of the target stimulus were randomized.

#### 2.2.2. Go/No-go Task

The Go/No-go task was implemented to measure the participants' ability to inhibit a prepotent response and to sustain their attention. For this task, letters of the alphabet were presented in an event-related design. Each trial started with the presentation of a single letter for 0.25 sec, followed by a variable poststimulus interval during which a fixation cross was visible. Trials lasted either 2, 6, or 8 sec with predominantly shorter trials (mean trial length = 3.5 sec). The participants' task was to respond to every letter (Go trials), except to the letter “X” (No-go trials), by pressing a key with their right index finger. They were instructed to focus on the fixation cross during the entire experiment and to respond as fast as possible and also to avoid mistakes. In total, 200 trials were presented with 35% No-go trials (“X”), and the complete task took 12 min. The sequence of letters as well as the presentation of No-go trials was randomized.

#### 2.2.3. Visual Task

We included a visual paradigm to test, in an explorative analysis, whether exercise-induced changes in brain activation were specific to executive task demands (i.e., constricted to executive task-related brain areas) or based on rather unspecific and general changes in neural activity. The visual task was presented at the beginning of the MRI session (T1) and, to test for the duration of potential exercise effects, again after the executive functioning tasks (T2). The stimuli were reversing checkerboard images adopted from Sandmann et al. [40]. The first image was a radial black and white checkerboard consisting of 20 rings that were divided into 18 sectors, where neighboring sectors were of opposite color. The second image was identical but rotated by 180°. Each image had a proportion of white pixels (luminance ratio) of 0.375. The diameter of the checkerboard was 10 cm, resulting in a visual angle of approximately 11°. The visual paradigm was presented in a block design that started with a block of pattern-reversing checkerboards for 30 sec, changing at a reversal rate of 3.3 Hz, followed by focusing a gray fixation cross for 20 sec. This sequence was presented three times so that the duration of the complete paradigm was 2.5 min.

### 2.3. Questionnaires

To evaluate participants' physical activity, they completed the German long form of the International Physical Activity Questionnaire (IPAQ-LF; http://www.ipaq.ki.se). The IPAQ is a self-report questionnaire that measures the subjective amount of physical activity during the last seven days. It consists of 27 items asking for the time (number of days and minutes/day) spent on walking and moderate and vigorous physical activities in four domains of daily life: work, transportation, domestic chores and gardening, and leisure time. Scoring was performed using the guidelines provided on the IPAQ website (http://www.ipaq.ki.se). For each domain and intensity level of physical activity, we computed MET-minutes per week and an overall physical activity score. MET-minutes (Metabolic Equivalent of Task) are the minutes spent on an activity multiplied by the energy costs of the specific activity. To assess participants' level of verbal intelligence, the Multiple Choice Vocabulary Test (MWT-B) [41] was used.

### 2.4. Experimental Procedure

On three different days, subjects participated in a preexperimental session (maximal exercise test) and in two experimental sessions (exercise and control conditions). All sessions were spaced a minimum of two days to prevent aftereffects of exercising on subsequent sessions. The experimental sessions were counterbalanced in their sequence, with half of the participants performing the exercise first and the other half the control condition. Participants were asked to avoid any physical exercise on the test days.

#### 2.4.1. Maximal Exercise Test

To determine participants' individual maximal heart rate (HR_max_) and to assess their cardiorespiratory fitness, they performed a maximal exercise test on a stationary bicycle ergometer. Participants started cycling at 90 W, and every 3 min, the resistance was increased by 40 W. The test was ended when the participant was subjectively exhausted (unable to continue pedaling). The heart rate and oxygen consumption were continuously recorded by way of a chest strap heart rate monitor (Polar RCX5, Polar Electro Oy, Finland) and a spirometer (Oxycon Mobile, CareFusion, Heidelberg, Germany), respectively. At the end of the test, we registered the participant's HR_max_ and peak oxygen consumption (VO_2peak_ in mL/min/kg). To determine cardiorespiratory fitness, we transformed VO_2peak_ values into age- and gender-adapted percentiles (VO_2peak_ % ranking) based on normative data provided by the American College of Sports Medicine (ACSM) [42].

#### 2.4.2. Experimental Sessions

To familiarize with the MR environment and the experimental tasks and to ensure correct task performance, participants started the first session performing practice trials of the flanker and Go/No-go tasks inside the MR scanner. Afterwards, depending on the sequence of the two conditions, they completed either the exercise or the control condition.

In the exercise condition, moderate exercise consisted of 30 min of continuous cycling on an ergometer at 50-70% of the individual HR_max_, which was selected in accordance with the guidelines of the ACSM [42]. For high intensity, a standard high-intensity interval training (HIIT) protocol was applied by a professional spinning instructor. It consisted of a 5 min warm-up phase, followed by short bursts of high-intensity cycling interspersed with varied recovery times (21 min in total), and ended with 4 min of cool-down. Excluding the warm-up and cool-down periods, intensities remained >70% of individual HR_max_ during the whole routine. The heart rate was continuously recorded via a chest strap heart rate monitor (Polar RCX5; Polar Electro Oy, Finland) and controlled by the experimenter.

In the control condition, all participants watched the Movie for the Assessment of Social Cognition (MASC-MCk) [43], which contains short sequences about four young characters spending an evening together for a dinner. The movie is about everyday social interactions and is paused after predefined sequences for questions about the actors' thoughts, feelings, and intentions. Participants are required to perform in a multiple-choice format to choose the correct one out of four possible answers. The MASC was chosen to keep participants engaged in a task that is entertaining but cognitively not demanding. Participants completed the movie after approximately 30 min.

Subsequently, in each condition, participants entered the MR scanner and followed the same procedure. First, the visual paradigm was presented (T1); afterwards, they performed the flanker task and the Go/No-go task; and at the end of the session, the visual paradigm was presented again (T2), and a structural scan was acquired.

### 2.5. fMRI Data Acquisition

Imaging data was acquired in a 3-Tesla MRI Scanner (Siemens MAGNETOM Verio, Siemens AG, Erlangen, Germany) with a 12-channel head array (*n* = 5 in each group) and a 3-Tesla MRI Scanner (Siemens Magnetom Prisma) with a 64-channel head array (*n* = 27 in each group). In each case, pairs of participants matched for age and gender were tested in the same scanner. To minimize head movements, foam pads were used. During the experimental tasks, functional images with BOLD contrast were obtained using multislice T2∗-weighted gradient echo planar imaging (EPI) (time of repetition (TR) = 1750 ms, time of echo (TE) = 30 ms, flip angle (FA) = 80°, Field of View (FoV) = 200 × 200 mm^2^, voxel size = 3.0 mm^3^, and matrix size: 64 × 64). Each experimental task was measured during a separate run. For the flanker task, 350 EPI volumes were measured, for the Go/No-go task 390 EPI volumes, and for each visual task 95 EPI volumes. Each EPI volume consisted of 31 3 mm thick axial slices, which were acquired sequentially with a 1 mm gap in an ascending order. Each volume covered the whole brain with the exception of the lowermost part of the cerebellum. After functional scanning, a high-resolution T1-weighted structural image using magnetization prepared rapid gradient-echo (MPRAGE) sequence (1 mm^3^ isotropic voxels, 176 slices, FoV = 250 × 250 mm, TR = 1900 ms, TE = 2.52 ms, and FA = 90°) was acquired.

### 2.6. Data Analysis

#### 2.6.1. Behavioral Analysis

Behavioral data analysis was conducted using RStudio 3.2.2 [44] and SPSS Statistics 22 (IBM, Armonk, NY, USA).

Flanker task trials were categorized into three types: congruent, incongruent, and neutral. For correct trials of each trial type, mean reaction times were calculated. Interference scores were computed by subtracting mean reaction time in congruent trials from mean reaction time in incongruent trials (RT incongruent − RT congruent). In addition, response accuracy as reflected by the proportions of errors and omissions was calculated.

Go/No-go task trials were classified into four types: hits (correct responses to non-X letters) and omissions (missed responses to non-X letters), as indicators of the ability to sustain attention over time, and correct inhibitions (correct withholding of responses to the X letter) and false alarms (incorrect responses to the X letter), as indicators of the ability to inhibit a prepotent response. To measure accuracy, proportion of hits (hit rate = number_hits_/number_Go trials_) and correct inhibitions (correct inhibition rate = number_correct inhibitions_/number_No−go trials_) were computed for each subject. Based on the signal detection theory [45], the sensitivity index *d*′ was calculated (*d*′ = *Z*(hit rate) − *Z*(false alarm rate)). This is a bias-free measure of the subject's ability to discriminate targets (signal) from distractors (noise). Finally, mean reaction times for hits were computed.

Our primary hypothesis was that moderate exercise would lead to greater improvements in performance than high-intensity exercise. Therefore, we calculated the differences in behavioral performance between the two conditions (exercise − control) and compared those between the two groups (moderate-intensity vs. high-intensity) using two-sample *t*-tests. To test for differences between conditions in each group separately, paired *t*-tests were used. Associations between behavioral outcomes and participants' fitness levels (VO_2peak_ % ranking values) were assessed using Pearson correlations. In cases where we had a clear hypothesis regarding the direction of exercise effects, one-sided tests were applied; in all other cases (i.e., effects of high-intensity exercise and correlation to fitness), two-sided tests were used.

As primary outcome measures, we defined the interference score for the flanker task and the sensitivity index *d*′ for the Go/No-go task. For those two measures, Bonferroni correction was used to account for multiple comparisons (significance level of *p* ≤ 0.025). As the interference score and the sensitivity index are summary measures and exercise may alter several components of executive functioning (e.g., attention and inhibition processes) as well as general arousal, we additionally analyzed the single components of those measures (i.e., RT congruent and RT incongruent for the flanker task, hit rate and correct inhibition rate for the Go/No-go task). Those analyses and those including fitness were exploratory, not confirmatory, and were interpreted accordingly. Therefore, no adjustment for multiple testing was applied (significance level of *p* ≤ 0.05).

#### 2.6.2. fMRI Analysis

MRI data were preprocessed and analyzed using SPM12 (Wellcome Trust Centre for Neuroimaging, London, UK) and Matlab R2016a. Preprocessing steps were (i) spatial realignment of the functional images to the mean image, (ii) coregistration of the T1-weighted structural image and the functional images, (iii) segmentation of the structural image, (iv) normalization to the standard MNI template brain (Montreal Neurological Institute, Quebec, Canada), and (v) smoothing using a 3D Gaussian kernel of 8 mm full-width-half-maximum (FWHM).

Statistical analyses on the single-subject level were run using the general linear model (GLM) in SPM12. For the flanker and Go/No-go tasks, we performed event-related analyses. To model task-related signal increases, stick functions that were time-locked to the event onsets were implemented. For the flanker task, the first-level GLM design matrix included five regressors of interest (correct congruent, correct incongruent, correct neutral, errors, and omissions), and the six head movement parameters were entered as nuisance regressors. The contrast *incongruent* − *congruent* representing brain activity specific to interference suppression as well as *congruent* and *incongruent* trials separately were of primary interest. For the Go/No-go task, the design matrix included four regressors of interest (hits, correct inhibitions, false alarms, and omissions), and six head movement parameters as nuisance regressors. The contrasts *hits*, *correct inhibitions*, and *correct* *inhibitions* − *hits* were of primary interest here, as they measure sustained attention and response inhibition. For the visual task, hemodynamic responses to watching pattern-reversing checkerboards were modeled as box-car functions with a block length of 30 sec convoluted with the HRF, time-locked to visual stimulation onsets. The GLM design matrix contained one regressor of interest, which modeled epochs of visual stimulation against an implicit baseline. Again, the six head movement parameters were entered as nuisance regressors. A high-pass filter of 1/128 Hz was applied to the fMRI time series in order to remove nonphysiological low-frequency noise. Temporal autocorrelation across scans was corrected using an AR(1) model. GLM parameters were estimated using the classical SPM approach, i.e., restricted maximum likelihood.

For each contrast of interest, we calculated differential contrasts comparing brain activation between the two conditions (*exercise* − *control* and *control* − *exercise*). On the group level, those differential contrasts were entered into two-sample *t*-tests to test for differences in condition-specific activation changes between the moderate and the high-intensity group. To examine within-group changes between the two conditions, we calculated paired *t*-tests using the contrasts of the single conditions for each group separately. To test for an association between cardiorespiratory fitness and exercise effects, we performed correlational analyses between VO_2peak_ % ranking values and differences in brain activation between the two conditions in the moderate and high-intensity group separately. The initial voxel threshold was set to 0.001 uncorrected. Multiple testing was then accounted for on the cluster level based on a corrected pFWE of 0.05. Peak coordinates are given in MNI space, and figures are displayed according to neurological convention.

#### 2.6.3. Gender Differences

To test whether exercise effects were modulated by gender, we compared exercise-related changes in the main behavioral and neural outcome measures between males and females using two-sample *t*-tests.

## 3. Results

### 3.1. Demographic Data

Participant characteristics and test scores are reported in Table 1. Groups did not differ with respect to age, gender, body mass index, cardiorespiratory fitness (VO_2peak_), subjective assessment of physical activity, or intelligence level.

### 3.2. Timing of Task Administration

After completion of the exercise protocol, the average time interval until the beginning of the experimental tasks was 6.2 min (SD = 1.6, range 4-11) for the visual task at T1, 10.1 min (SD = 2.3, range 7-18) for the flanker task, 21.4 min (SD = 2.6, range 18-29) for the Go/No-go task, and 33.6 min (SD = 2.8, range 30-41) for the visual task at T2.

### 3.3. Behavioral Results

**Figure 1:**
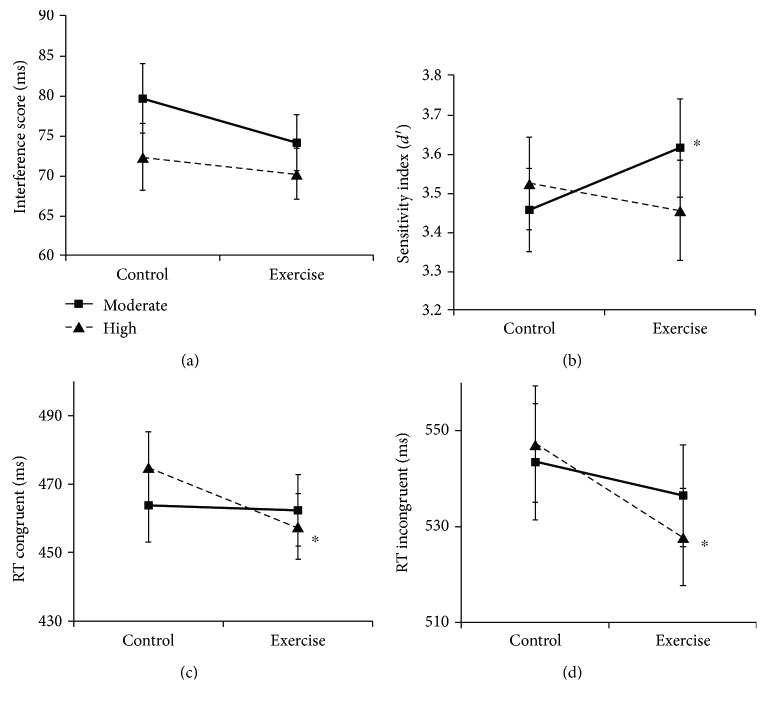
Exercise effects on primary outcome measures of the two executive functioning tasks ((a) flanker task and (b) Go/No-go task) and measures found to be influenced by high-intensity exercise in an explorative analysis: reaction times in (c) congruent and (d) incongruent trials of the flanker task. ^∗^ *p* < 0.05 (refers to pairwise comparisons, i.e., paired *t*-tests). (Mean values with standard error).

Table 2 shows the group means for all behavioral measures. Main behavioral results are presented in Figure 1.

#### 3.3.1. Flanker Task

For the interference score (RT incongruent − RT congruent), which was the primary outcome measure of the flanker task, we neither found differences in exercise-related changes between the moderate- and high-intensity groups nor changes between the two conditions in the single groups (*p* > 0.025). However, an explorative analysis for the high-intensity group revealed that reaction times in both congruent and incongruent trials improved in the exercise condition compared to the control condition (paired *t*-tests; congruent: *t*(30) = −2.70, *p* = 0.011, *d* = 0.48; incongruent: *t*(30) = −3.12, *p* = 0.004, *d* = 0.56; two-sided).

#### 3.3.2. Go/No-go Task

The moderate-intensity group compared to the high-intensity group showed greater exercise-related changes in the sensitivity index *d*′ (*t*(61) = 2.96, *p* = 0.045, *d* = 0.43), which did, however, not survive Bonferroni correction (significance level of *p* ≤ 0.025). Exploratory paired *t*-tests showed an increase in *d*′ in the exercise condition compared to the control condition in the moderate-intensity group (*t*(31) = 2.04, *p* = 0.025, *d* = 0.36). No significant effects were obtained for the hit rate or correct inhibition rate (*p* > 0.05).

### 3.4. fMRI Results

Our main interest was related to differences in brain activation between the exercise and control conditions. However, to test whether our executive functioning tasks evoked brain activation patterns similar to those reported in previous studies, we first examined brain activation specific to interference control (flanker task) and response inhibition (Go/No-go task) for the control condition only. In both tasks, we observed activations in frontal areas (including the inferior frontal gyrus and the insula) as well as in sensorimotor regions, replicating findings described in previous literature. These results are described in Supplementary Materials (available here).

#### 3.4.1. Flanker Task

There were neither differences in exercise-related activation changes between the two groups nor within-group effects of condition for any contrast of interest.

#### 3.4.2. Go/No-go Task

**Figure 2:**
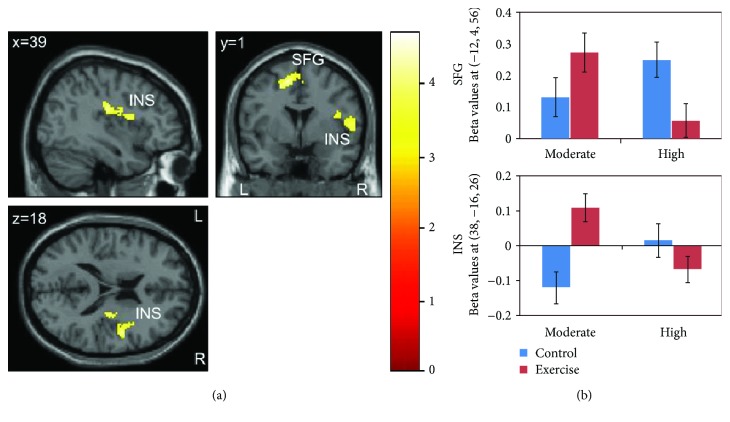
Brain activation during the Go/No-go task for the contrast *hits*: (a) activation specific to *moderate* (*exercise* − *control*)–*high* (*exercise* − *control*); (b) mean beta values of peak coordinates with standard error for each group and condition separately. Activation differences were found in two clusters with peak activation in the left superior frontal gyrus (SFG) and right insula (INS). *p* < 0.05 (FWE-corrected on cluster level, initial voxel threshold 0.001 uncorrected).

We found significant differences in exercise-related changes in brain activation between the two groups for correct responses in Go trials (contrast *hits*) in two clusters (Figure 2(a)): the first cluster was located in the left superior frontal gyrus and included the supplementary motor area and parts of the presupplementary motor area; the second cluster presented with a peak activation in the right insula and extended to the precentral gyrus and slightly to the inferior frontal gyrus. BOLD responses within those clusters increased in the moderate-intensity group and decreased in the high-intensity group in the exercise condition compared to the control condition (Figure 2(b)). Peak coordinates and statistical results are given in Table 3.

Paired *t*-tests revealed that, in the moderate-intensity group, brain activation during *hits* increased in the exercise condition compared to the control condition in three clusters (Table 3): the first cluster was located in the left superior and middle frontal gyri; the second cluster extended from the right precentral gyrus to the inferior frontal operculum, the rolandic operculum, and the insula; the third cluster comprised the left inferior frontal gyrus (pars triangularis), inferior frontal operculum, and parts of the middle frontal gyrus. In the high-intensity group, there was no significant difference in brain activation between the two conditions. No other contrasts of interest reached statistical significance neither between nor within groups.

#### 3.4.3. Visual Task

**Figure 3:**
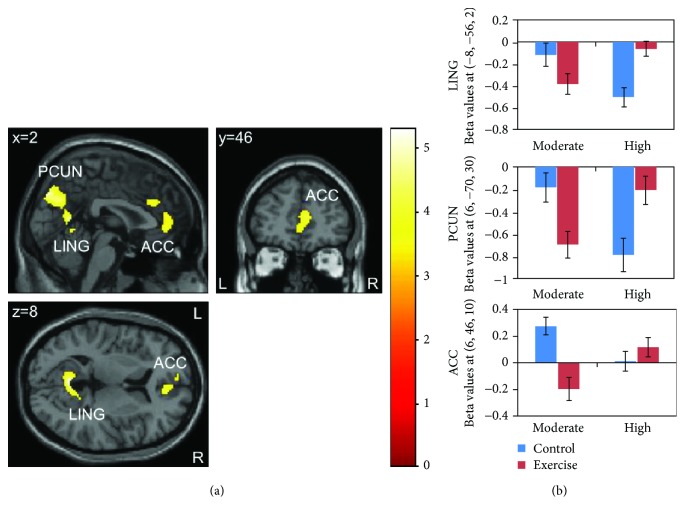
Brain activation during visual stimulation: (a) activation specific to *high* (*exercise* − *control*)–*moderate* (*exercise* − *control*); (b) mean beta values of peak coordinates with standard error for each group and condition separately. Activation differences were found in three clusters with peak activation in the lingual gyrus (LING), precuneus (PCUN), and anterior cingulate cortex (ACC). *p* < 0.05 (FWE-corrected on cluster level, initial voxel threshold 0.001 uncorrected).

For the visual task presented directly after each condition (T1), we found a difference in exercise-related activation changes between the moderate- and high-intensity groups in three clusters located in the midline structures (Figure 3(a)): the first cluster had a peak activation in the lingual gyrus and comprised the calcarine sulcus and cuneus; the second cluster was located in the precuneus and extended to the cuneus, the superior occipital gyrus, and the cerebellum; the third cluster extended from the anterior cingulate cortex to the midcingulate cortex. BOLD responses within those clusters showed decreased activation in the moderate-intensity group and increased activation in the high-intensity group in the exercise condition compared to the control condition (Figure 3(b)). Peak coordinates and statistical results are described in Table 4.

Paired *t*-tests showed that, in the moderate-intensity group, brain activation during visual stimulation significantly decreased in the exercise condition compared to the control condition in one cluster with peak activation in the anterior cingulate cortex, extending to the midcingulate cortex and the medial part of the orbitofrontal cortex (Table 4). In contrast, brain activation in the high-intensity group increased in the exercise condition in two clusters: the first cluster comprised parts of the lingual gyrus, the vermis, and the calcarine sulcus; the second cluster was located in the precuneus and extended to the superior occipital gyrus and the cuneus (Table 4).

For the visual task at the end of the scanning session (T2), there were neither differences in exercise-related activation changes between the two groups nor condition-specific differences in the single groups.

### 3.5. Association between Cardiorespiratory Fitness and Exercise Effects

#### 3.5.1. Flanker Task

**Figure 4:**
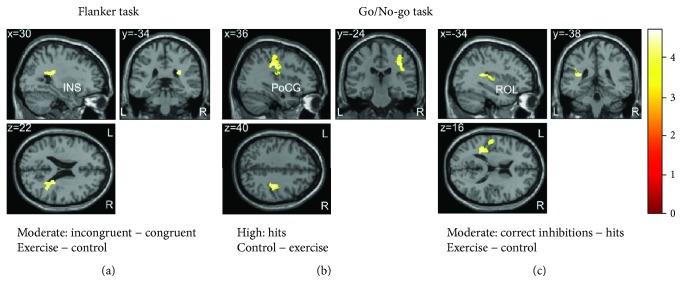
Correlations between cardiorespiratory fitness (VO_2peak_ % ranking values) and changes in BOLD responses between the two conditions. (a) Flanker task: in the *moderate-intensity* group, fitness correlated positively with differential BOLD response (*exercise* − *control*) for the contrast *incongruent* − *congruent* in one cluster with peak activation in the right insula (INS). (b) Go/No-go task: in the *high-intensity* group, higher fitness was associated with stronger exercise-related decreases in BOLD response (*control* − *exercise*) for the contrast *hits* in one cluster with peak activation in the right postcentral gyrus (PoCG). (c) Go/No-go task: in the *moderate-intensity* group, fitness correlated positively with differential BOLD response (*exercise* − *control*) for the contrast *correct* *inhibitions* − *hits* in one cluster with peak activation in the left rolandic operculum (ROL). *p* < 0.05 (FWE-corrected on cluster level, initial voxel threshold 0.001 uncorrected).

In the moderate-intensity group, we found a positive correlation of VO_2peak_ with differential BOLD response (*exercise* − *control*) for the contrast *incongruent* − *congruent* in one cluster which was located in the right insula and extended to the rolandic operculum and superior temporal gyrus ((*x*, *y*, *z*) = (30, −34, 22), *k* = 178; Figure 4(a)). VO_2peak_ did not correlate with behavioral outcomes.

#### 3.5.2. Go/No-go Task

In the high-intensity group, brain activation during *hits* in the exercise condition compared to the control condition negatively correlated with VO_2peak_. In other words, higher fit participants showed stronger exercise-related decreases in brain activation in one cluster comprising the right postcentral gyrus, rolandic operculum, supramarginal gyrus, and insula ((*x*, *y*, *z*) = (36, −24, 40), *k* = 506; Figure 4(b)). In the moderate-intensity group, VO_2peak_ did not correlate with changes in brain activation during *hits*, but we observed a significant positive correlation with changes in BOLD responses for the contrast *correct* *inhibitions* − *hits* in one cluster comprising the left rolandic operculum, insula, postcentral gyrus, Heschl's gyrus, and supramarginal gyrus ((*x*, *y*, *z*) = (−34, −38, 16), *k* = 427; Figure 4(c)). Participants with higher fitness showed stronger increases in brain activation in the exercise condition compared to the control condition in this cluster. We found no significant correlation of VO_2peak_ with behavioral outcomes in the Go/No-go task.

#### 3.5.3. Visual Task

Cardiorespiratory fitness did not correlate with changes in brain activation during the visual tasks in neither group.

### 3.6. Gender Differences

For hit trials of the Go/No-go task, males in the moderate-intensity group showed greater exercise-related increases in brain activation than females in one cluster with peak activation in the right precentral gyrus, extending to the supramarginal and middle frontal gyri. Comparing condition-specific activation changes (*exercise*−*control*) during *hits* between male participants of the moderate- and high-intensity groups, we found differences in six clusters comprising frontal, parietal, and temporal regions (e.g., insula, inferior frontal gyrus, and supplementary motor area). Results of those analyses are described in more detail in Supplementary Materials.

## 4. Discussion

The primary objective of the current study was to investigate intensity-dependent effects of acute exercise on executive functioning in healthy adults. Executive performance during the Go/No-go task tended to improve to a greater extent by moderate-intensity exercise compared to high-intensity exercise. In addition, moderate exercise was associated with increased brain activation in task-related areas during the Go/No-go task. In contrast, high-intensity exercise was not associated with changes in behavioral performance but with decreased activation in task-related brain areas. Other than expected, exercise at moderate intensity had no effects on the primary behavioral outcome measure of the flanker task. However, an explorative analysis revealed that high-intensity exercise was associated with faster reaction times, which might indicate improved attention and processing speed. Effects of exercise on brain activation patterns were observed for none of the exercise intensities in this task. A second hypothesis was related to cardiorespiratory fitness, postulating an association with intensity-dependent exercise effects. Noteworthy, cardiorespiratory fitness was correlated with exercise effects on brain activation in both groups, while the direction of the correlation was dependent on exercise intensity. Higher fitness was associated with increased brain activation in task-related areas after moderate exercise and decreased brain activation after high-intensity exercise.

### 4.1. Exercise-Induced Behavioral Changes

Exercise at different intensities affected performance in the two executive tasks differently. Moderate exercise improved Go/No-go task performance (i.e., sensitivity index *d*′), although we found no effects on our primary outcome measure for the flanker task (i.e., interference score). The finding of improved Go/No-go task performance after moderate exercise is in line with our hypothesis and with previous literature reporting positive effects of moderate-intensity exercise on executive functioning [3, 4, 8, 46, 47]. Several reviews and meta-analyses also reported that exercise at moderate intensity may be most beneficial for executive functioning [3, 4, 8]. Note that behavioral effects of moderate exercise were less pronounced than expected, i.e., after Bonferroni correction, only a tendency was observed, which could be attributed to already high performance levels in the control condition. Previous reviews and meta-analyses demonstrated that persons showing lower performance levels or undergoing neurophysiological changes (e.g., patients, children, or older adults) are more susceptible to benefit from exercise than young adults [2, 30, 48]. We would therefore expect more pronounced enhancements in those groups.

Exercise at high intensity had no influence on the primary outcome measures of the executive tasks in our study, but on an exploratory basis, we observed improved reaction times during congruent and incongruent trials of the flanker task. Note that previous results on effects of high-intensity exercise are highly heterogeneous [3]. One study examining the long-term effects of moderate and high-intensity exercise on older individuals' cognitive functions reported that moderate-intensity exercise was superior in the enhancement of executive functions while high-intensity exercise was most beneficial for information processing speed in the Stroop task [49]. These results are in line with our findings of improved reaction times in the flanker task after high-intensity exercise, indicating improved processing speed and attention, possibly caused by increased arousal, rather than improved interference control.

As we had hypothesized the same pattern of behavioral changes for both executive function tasks, the lack of moderate exercise effects on performance in the flanker task was surprising. Both tasks measure slightly different components of inhibition, namely, interference control (i.e., suppression of distractors and competing responses) and selective attention for the flanker task and response inhibition (which involves the control of primary motor responses) and sustained attention for the Go/No-go task [1, 50]. Therefore, the unique effect of moderate exercise on Go/No-go task performance could imply that only certain aspects of inhibitory processes are affected by moderate exercise. However, some of the previous studies have reported exercise-related improvements in flanker task performance even though a few studies have found no effects [3].

Another explanation for the different outcomes in the two tasks might be the difference in timing. Task performance occurred from around 10 to 20 min after exercise completion for the flanker task and from around 21 to 33 min after exercise completion for the Go/No-go task. Unfortunately, in many studies, exact timing of the tasks is not reported, and literature regarding the time course of exercise effects is scarce and inconsistent. While Joyce et al. [46] have claimed that the benefits of moderate exercise remain present for up to 52 min, others have argued that improvement in cognitive performance lasts shorter than 30 min following exercise cessation [47]. One meta-analysis concluded that cognitive tests administered 11-20 min after exercise result in larger effects compared to those administered directly after exercise. In addition, the effects were smaller after 20 min following exercise completion [2]. Contrary to these findings, improvements in executive functioning in our study were only present in the second task (after around 21 min following moderate exercise), while we found improved reaction times in the first task after high-intensity exercise. With respect to the time course of exercise effects, these results might be explained by a strong initial increase in arousal after high-intensity exercise probably followed by a more exhausted state after around 20 min. After moderate exercise, arousal might have been less affected, resulting in a different time course of exercise effects on task performance. In addition, task sequence was not counterbalanced in this study, which might have also influenced outcomes. When performing long-lasting cognitive demanding tasks, fatigue and consequently a decrease in attention might occur. Acute exercise could counteract those processes, which might explain why exercise effects were only found for the second task.

### 4.2. Exercise-Induced Brain Activation Changes

We found intensity-dependent effects of exercise on brain activation patterns during the Go/No-go task, with a significant difference in exercise-related activation changes between the two groups for successful Go trials (hits). Compared to the control condition, exercise at moderate intensity was associated with increased brain activation while exercise at high intensity was associated with decreased brain activation in task-related areas. The fact that these changes were observed during hits rather than successful No-go trials (correct inhibitions) indicates exercise-induced changes in vigilance and sustained attention rather than in response inhibition per se.

Brain activation changes associated with exercise were observed in two clusters of the frontal lobe. The first cluster comprised medial parts of the premotor cortex including the supplementary motor area (SMA), more lateral parts of the superior frontal gyrus, and parts of the pre-SMA. The premotor cortex has previously been shown to be involved in executive function tasks, predominantly responsible for preparation, selection, and execution of motor responses [51–56]. The SMA in particular has been associated with response execution, motor planning, and resolution of response conflict [1, 57, 58] and has been found to be active during Go trials in previous studies using a similar paradigm [56, 59, 60]. As distinct from the more posterior SMA, which is connected to primary motor regions, the pre-SMA is mainly connected to prefrontal regions. Numerous studies have highlighted its importance for cognitive control, specifically when motor actions need to be inhibited (see reviews by [53, 61]). The second cluster was located in the right insula extending to the precentral gyrus, which is part of the lateral premotor cortex, and slightly ranging into the right inferior frontal gyrus. In addition, moderate exercise was associated with increased activation in a third cluster located in the left inferior frontal gyrus (paired *t*-test). Several reviews suggest that the insula and right inferior frontal cortex together with the pre-SMA form a network that is activated during tasks of response inhibition [62, 63]. While the right inferior frontal cortex is consistently considered to be critical for response inhibition (e.g., [64]), the role of the left inferior frontal cortex is less clear. Nevertheless, some studies have demonstrated that also the left inferior frontal gyrus plays an important role in response inhibition (e.g., [65, 66]). The insula has also been implicated in the maintenance of task rules and readiness [67], in tasks of attention and saliency [68], and in mechanisms of response selection [69].

Regions such as the insula and the SMA belong to a task-positive network (i.e., show increases in activation during cognitive demanding tasks) [70]. Considering the tendency towards improvements in behavioral task performance specific to exercise at moderate intensity, the finding of increased brain activation after moderate exercise possibly indicates enhanced cognitive and motor processing. On the other hand, high-intensity exercise led to decreases in brain activation, which could indicate fatigue. However, in light of the lack of changes in behavioral performance, the brain activation changes associated with high-intensity exercise are difficult to interpret. Nevertheless, our results seem to point into the direction of the inverted-U hypothesis proposing that neurophysiological changes and cognitive performance are optimal for moderate-intensity exercise, while high-intensity exercise would lead to neural noise [16, 17]. No exercise-related changes in brain activation were observed during the flanker task, which is contrary to our hypothesis but in accordance with the lack of changes in behavioral task performance after moderate exercise. Again, these findings could indicate that only certain aspects of executive function benefit from moderate exercise. Alternatively, the timing or sequence of tasks could be an explanation. The time course of neurophysiological changes due to exercise in particular is largely unknown.

The current study is the first to use fMRI to examine the impact of exercise at different intensities on executive functioning. Due to differences in study design, sample characteristics, and cognitive functions assessed, our results are difficult to compare to the few existing neuroimaging studies exploring the relationship between acute exercise and executive functions. MacIntosh et al. [20] tested the effects of moderate exercise on response inhibition in 16 healthy adults, also using a Go/No-go task. They reported decreased activation postexercise compared to preexercise in the left parietal operculum, combined with no behavioral changes. The study differed in design as no control condition was included, inducing the possibility of habituation effects. In addition, two different studies using fNIRS while subjects performed a Stroop task showed that cerebral oxygenation over the prefrontal cortex increased after moderate aerobic exercise [71] and decreased during vigorous aerobic exercise [72]. Contrary, Chang et al. [21] found decreased prefrontal cortex oxygenation during a Stroop task after high-intensity resistance exercise but not after high-intensity aerobic exercise. Note that the studies discussed above did not provide information on participants' fitness level, which might modulate the effects of exercise, in particular the effects of different exercise intensities.

### 4.3. Association between Cardiorespiratory Fitness and Exercise Effects

In our study, cardiorespiratory fitness as measured by VO_2peak_ was related to exercise-induced brain activation changes during executive function tasks in both intensity groups. Interestingly, the direction of correlations differed depending on exercise intensity. More specifically, in the high-intensity group, fitness was negatively correlated with brain activation changes during hit trials of the Go/No-go task. Participants with higher fitness levels showed stronger exercise-related activation decreases in a cluster comprising the right insula, rolandic operculum, and postcentral gyrus. This finding indicates that fitness may modulate the effects of high-intensity exercise, possibly due to interactions between fitness and neurophysiological changes induced by exercise.

In the moderate-intensity group, fitness was not related to brain activation changes during hit trials, indicating exercise effects on attentional processes independent of the fitness level. In contrast, we found a positive correlation of fitness with brain activation changes specific to response inhibition (contrast *correct* *inhibitions* − *hits*). Participants with higher fitness levels revealed stronger increases in brain activation after moderate exercise compared to the control condition in one cluster comprising the left rolandic operculum, insula, and postcentral gyrus. Similarly, we found a correlation of fitness with brain activation changes specific to interference control (contrast *incongruent* − *congruent*) as measured by the flanker task in the moderate-intensity group. Participants with higher fitness showed stronger increases in brain activation after moderate exercise in one cluster comprising the right insula, rolandic operculum, and superior temporal gyrus.

Taken together, those findings indicate that participants with higher fitness levels might profit to a greater extent from moderate exercise compared to participants with lower fitness levels. However, we found no associations between fitness and behavioral outcome measures. Further, it is worth to note that all analyses involving cardiorespiratory fitness were explorative and should be interpreted accordingly. Few studies so far have investigated the potential moderating role of fitness on effects of acute exercise on cognition. Among those, the majority have focused on behavioral measures (e.g., [29, 73]), while others have included EEG measures (e.g., [31, 32, 74, 75]). No previous fMRI studies have been conducted on that topic, and likewise, there is no study which investigated the influence of fitness on effects of different exercise intensities. Our findings provide first tentative evidence that fitness might have a moderating role on acute exercise effects at both moderate and high intensities.

### 4.4. Effects of Exercise on Brain Activation during the Visual Task

During visual stimulation directly following exercise cessation (T1), we observed a differential effect of exercise at different intensities on brain activation patterns in three clusters located in the midline structures: two clusters comprised parietal and occipital areas (e.g., lingual gyrus, cuneus, and precuneus); the third cluster was located around the anterior cingulate cortex. Participants revealed stronger deactivation in those clusters after moderate-intensity exercise as compared to the control condition. In contrast, after high-intensity exercise, we found weaker deactivation or increased activation in these clusters. This pattern of brain activation changes is opposite to the activation changes found for the Go/No-go task where brain activation was increased after moderate exercise and decreased after high-intensity exercise. However, whereas brain regions found to be active during the Go/No-go task belong to a task-positive network [70], exercise-related activation changes during visual stimulation occurred in brain areas that are part of a task-negative network, the so-called default mode network [76]. Regions of this network are highly functionally interconnected and show strongly correlated activations during rest [77], whereas during task performance, activation in those areas decreases [76, 78, 79]. Activation of the default mode network has been linked to internal mental processing, the occurrence of thoughts independent of external stimuli, self-focused attention, and representations of states of the self [78, 80, 81]. On the other hand, deactivation of the default mode network has been related to non-self-referential goal-directed behavior and the allocation of attentional resources to an external input [76, 78].

Accordingly, we propose that stronger deactivation of regions of the default mode network (i.e., anterior cingulate cortex and precuneus) after moderate exercise might imply enhanced allocation of attention to the checkerboard stimuli, which matches the positive effects of moderate exercise on Go/No-go task performance and related brain activation patterns. The finding of weaker deactivation in the high-intensity group could indicate impaired attention to the visual stimulus, possibly due to heightened attention to the internal state (increased physiological processes) immediately after intense exercise. This matches the finding of decreased brain activation in task-positive areas during Go/No-go task performance. A putative neurophysiological explanation implies an increase of arousal which might be at an optimal level after moderate exercise whereas a further increase (i.e., due to high-intensity exercise) might lead to impairments in the control of cognitive resources, hence following the inverted-U shape [16, 17].

Literature on the effects of exercise on visual processing is scarce. A handful of EEG studies found that different components of the visual evoked potential can be modified by exercise. For instance, exercise reduced the latency and modified the amplitude of the P100, N145, and P300 components of the visual evoked potential, indicating an effect of exercise on visual processing speed and visual attention [82–85]. Authors concluded that different stages of stimulus processing, i.e., early perceptual processes as well as late attentional processes, can be influenced by exercise [86, 87]. Specifically, the P100 component has been demonstrated to be modulated depending on the amount of attentional resources allocated to the stimulus [86, 88]. One study examined visual evoked potentials after exercise at different intensities (40%, 65-75%, and 80% of VO_2max_) compared to rest in athletes and nonathletes [85]. Only nonathletes showed a reduced latency of the P100 wave (related to processing of visual stimuli) following low and moderate intensities. After intense exercise, however, P100 latency increased and P100 amplitude decreased. In athletes, these parameters did not change despite the increases in exercise intensity. With respect to cardiorespiratory fitness, we found no correlation between fitness and exercise-induced changes in brain activation during visual stimulation, indicating that the proposed increase in arousal observed shortly after exercise occurs irrespective of fitness. In the ongoing time course of neurophysiological processes, fitness might play a more important role, in particular when it comes to cognitive demanding performance (i.e., executive tasks).

The visual task was included to test whether exercise-related changes in brain activation were specific to executive task demands or based on rather unspecific and general changes in neural activity. Our results indicate an effect of exercise on neural activity that is not restricted to executive tasks. Interestingly, rather than unspecific changes, we observed changes in a brain network that is highly interconnected and known to be deactivated during tasks. During visual stimulation at T2 (approximately 34 min after exercise), there was no difference in brain activation between the exercise and control conditions, indicating that exercise-induced effects did not last for more than 30 min, regardless of exercise intensity.

### 4.5. Strengths and Limitations

One strength of the current study is the inclusion of a control condition which was conducted on a different day, while the order of the exercise and control conditions was randomized and counterbalanced to avoid practice effects. Second, in contrast to most previous studies, in which exercise intensity was calculated based on estimated HR_max_, we performed a maximal exercise test to assess the individual HR_max_. Third, this is the first fMRI study considering cardiorespiratory fitness as a factor which has a potential influence on acute exercise effects. Nonetheless, a limitation is that we recruited and allocated participants to the two groups irrespective of their fitness level. Although mean VO_2peak_ did not differ between groups, there was a difference in the distribution of fitness levels. In the high-intensity group, only one third of the participants could be classified as showing a high degree of fitness (VO_2peak_ > 50), whereas in the moderate-intensity group, participants with high and low fitness were approximately equally distributed. To further assess the moderating role of fitness on exercise effects, future studies should systematically recruit participants from the full spectrum of fitness levels. In addition, fMRI data acquisition was conducted on two different MR scanners using two different head coils, which can produce changes in the temporal signal-to-noise ratio. As only five participants in each group were tested with a 12-channel head coil, we conducted all fMRI analyses again excluding those participants, yielding very similar results to those obtained for the complete group.

## 5. Conclusions

This study is the first to show that exercise at moderate intensity vs. high intensity has different effects on executive task-related brain activation as measured by fMRI. Our results in some aspects confirm previous findings of beneficial effects of acute moderate exercise on executive functioning and extend the literature on high-intensity exercise effects, indicating improvements in processing speed and attention. In addition, we could contribute to clarifying the underlying neurophysiological mechanisms of exercise benefits on cognition. Our data suggest exercise effects on neural activity that are not restricted to executive tasks. Further, cardiorespiratory fitness and type and timing of cognitive task possibly are important factors to consider when testing exercise effects. Results of this study might also encourage investigation of exercise effects in groups showing lower performance levels or undergoing neurophysiological changes.

## Figures and Tables

**Table 1:** Demographic and clinical characteristics of participants in the moderate-intensity and high-intensity groups.

Variable	Moderate intensity (mean ± SD)	High intensity (mean ± SD)	*t*-statistic^1^	*p* value
Age (years)	29.3 ± 8.5	28.6 ± 7.7	0.33	0.74
BMI (kg/m^2^)	23.8 ± 2.3	24.5 ± 4.8	-0.73	0.47
HR_max_ (beats/min)	185.3 ± 11.0	183.3 ± 11.1	0.69	0.49
VO_2peak_ (mL/min/kg)	39.6 ± 7.1	37.0 ± 8.3	1.34	0.19
VO_2peak_ (% ranking)	48.4 ± 19.9	40.2 ± 21.7	1.58	0.12
BDI	4.0 ± 5.3	5.5 ± 5.7	-1.07	0.29
MWT-B	29.1 ± 4.4	28.8 ± 5.7	0.23	0.82
PA total score	4495 ± 3852	5133 ± 4538	-0.60	0.55
PA work	827 ± 2465	1324 ± 2790	-0.75	0.46
PA transportation	1194 ± 971	1038 ± 1126	0.59	0.56
PA domestic	975 ± 1982	1130 ± 2236	-0.29	0.77
PA leisure	1499 ± 1601	1642 ± 2069	-0.31	0.76
PA walking	721 ± 878	922 ± 1177	-0.77	0.44
PA moderate	2513 ± 2453	2332 ± 2451	0.29	0.77
PA vigorous	1261 ± 2241	1879 ± 2551	-1.02	0.31

^1^Two-sample *t*-test; *df* = 61. Moderate intensity: *N* = 32 (16 females); high intensity: *N* = 31 (16 females). SD = standard deviation; BMI = body mass index; HR_max_ = maximal heart rate as assessed by maximal exercise test; VO_2peak_ = peak oxygen consumption as tested by the maximal exercise test; VO_2peak_ (% ranking) = peak oxygen uptake transformed into age- and gender-adapted percentiles; BDI = Beck Depression Inventory; MWT-B = Multiple Choice Vocabulary Test; PA = physical activity as assessed by the International Physical Activity Questionnaire, expressed in MET-minutes/week.

**Table 2:** Behavioral performance during the executive function tasks for each group and condition.

Variable	Moderate intensity		High intensity	
	Control mean (SE)	Exercise mean (SE)	Control mean (SE)	Exercise mean (SE)
*Flanker task*				
Interference score (ms)	80 (4)	74 (3)	72 (4)	70 (3)
RT congruent (ms)	464 (11)	462 (10)	475 (10)	458 (10)
RT incongruent (ms)	543 (12)	536 (11)	547 (12)	528 (10)
Error rate	0.029 (0.004)	0.027 (0.004)	0.023 (0.003)	0.025 (0.004)
Omission rate	0.006 (0.002)	0.005 (0.002)	0.007 (0.004)	0.003 (0.018)
*Go/No-go task*				
Sensitivity index (*d*′)	3.45 (0.11)	3.61 (0.13)	3.52 (0.12)	3.45 (0.13)
Hit rate	0.974 (0.009)	0.976 (0.007)	0.981 (0.007)	0.964 (0.015)
Correct inhibition rate	0.918 (0.014)	0.930 (0.017)	0.914 (0.012)	0.915 (0.014)
RT hits (ms)	517 (11)	514 (11)	531 (14)	525 (14)

RT = reaction time; SE = standard error of the mean.

**Table 3:** Brain activation during hit trials of the Go/No-go task.

Group, condition	Region of peak activation	MNI coordinates (*x*, *y*, *z*)	Cluster size	*t*-statistic	*z*-statistic	*p* ^∗^
MI (exercise − control)–HI (exercise − control)^1^	L superior frontal	-12, 4, 56	314	4.65	4.28	0.020
	R insula	38, -16, 26	484	4.44	4.12	0.003

MI (exercise − control)^2^	L superior frontal	-20, 4, 50	366	5.57	4.60	0.009
	R precentral	44, 2, 26	409	5.16	4.35	0.005
	L inferior frontal pars triangularis	-42, 26, 20	359	4.70	4.05	0.010

HI (control − exercise)^2^	Not significant					

^∗^FWE-corrected on cluster level (initial voxel threshold 0.001 uncorrected); ^1^two-sample *t*-test; ^2^paired *t*-test. MI = moderate intensity; HI = high intensity.

**Table 4:** Brain activation during the visual task presented directly after each condition (T1).

Group, condition	Region of peak activation	MNI coordinates (*x*, *y*, *z*)	Cluster Size	*t*-statistic	*z*-statistic	*p* ^∗^
HI (exercise − control)–MI (exercise − control)^1^	L lingual gyrus	-8, -56, 2	582	5.31	4.80	0.001
	R precuneus	6, -70, 30	1276	4.80	4.41	<0.001
	L anterior cingulate cortex	6, 46, 10	578	4.22	3.94	0.001

MI (control − exercise)^2^	L anterior cingulate cortex	6, 40, 14	1583	5.30	4.44	<0.001

HI (exercise − control)^2^	L lingual gyrus	-6, -54, 0	612	4.95	4.20	0.001
	Precuneus	0, -80, 42	267	4.40	3.83	0.030

^∗^FWE-corrected on cluster level (initial voxel threshold 0.001 uncorrected); ^1^two-sample *t*-test; ^2^paired *t*-test. MI = moderate intensity; HI = high intensity.

## Data Availability

The data used to support the findings of this study are available from the corresponding author upon request.

## Abbreviations

BDNF: Brain-derived neurotrophic factor
CBF: Cerebral blood flow
HR_max_: Maximal heart rate
VO_2peak_: Peak oxygen consumption as tested by maximal exercise test (in mL/min/kg)
RT: Reaction time
SMA: Supplementary motor area.

## Supplementary Materials

Supplementary Materials Task-related brain activation in the control condition as well as gender differences regarding exercise effects on brain activation are described.
