# Supplementary material for: Intensity-Dependent Effects of Acute Exercise on Executive Function
Source: Neural Plast. 2019 Jun 4;2019:8608317. doi: 10.1155/2019/8608317 (PMC6589258; doi:10.1155/2019/8608317)
Supplement: Supplementary Materials — Task-related brain activation in the control condition as well as gender differences regarding exercise effects on brain activation are described. [file 8608317.f1.docx]

**Supplementary Materials**

*Task-related brain activation within the control condition (after watching a movie)*

Fig. S1 shows brain activation specific to interference control (flanker task: contrast *incongruent - congruent*) and response inhibition (Go/No-go task: contrast *correct inhibitions – hits*) averaged over both groups within the control condition.

Participants showed brain activation related to interference control during the flanker task in five clusters: the first cluster comprised post- and precentral regions of both hemispheres, including the supplementary motor area ([x, y, z] = [-42, -34, 44], k = 9175); the second showed peak activation in the right middle temporal gyrus and extended to the right hippocampus, inferior temporal gyrus, supramarginal gyrus, and the inferior occipital gyrus ([x, y, z] = [48, -60, 4], k = 1660); the third was located in the left middle occipital gyrus extending to left inferior and middle temporal regions ([x, y, z] = [-50, -74, 4]; k = 908); the fourth comprised the right insula, nucleus caudatus, inferior frontal regions (operculum, pars triangularis), rolandic operculum, and putamen ([x, y, z] = [34, 24, 6], k = 896); the fifth cluster had peak activation in the left inferior frontal operculum and included the pars triangularis and orbital parts of the left inferior frontal gyrus as well as the insula ([x, y, z] = [-48, 4, 6], k = 1185).

Brain activation related to response inhibition during the Go/No-go task was observed in four clusters: the first cluster comprised the left insula, putamen, pars triangularis, and the orbital part of the inferior frontal gyrus ([x, y, z] = [-30, 16, -8], k = 573); the second showed peak activation in the right insula and extended to the right amygdala, pars triangularis and orbital part of the inferior frontal gyrus, and the precentral gyrus ([x, y, z] = [32, 26, 4], k =1982); the third included the right superior temporal gyrus and superior temporal pole, and parts of the angular gyrus and the insula ([x, y, z] = [52, -44, 14], k = 1275); the fourth was located in the right postcentral gyrus extending to the precentral gyrus ([x, y, z] = [38, -22, 50], k = 560).

a) Flanker task b) Go/No-go task

**
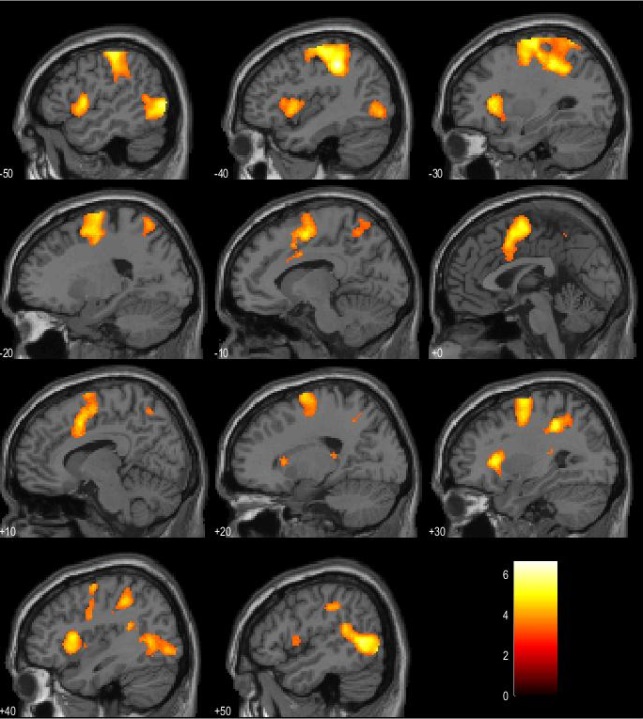
***
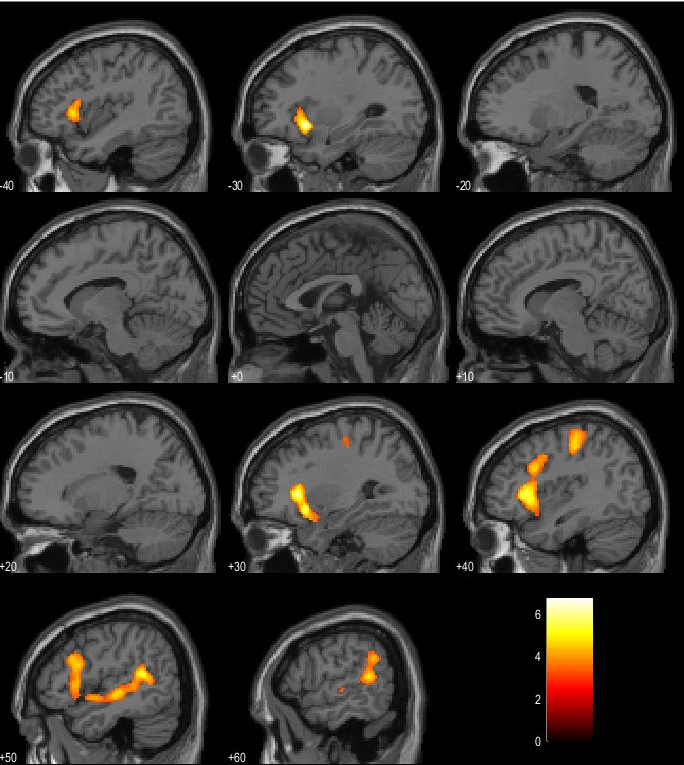
*

**Fig. S1**

Brain activation in the control condition averaged over both groups during a) flanker task for the contrast *incongruent – congruent* and b) Go/No-go task for the contrast *correct inhibitions – hits*. p<.05 (FWE-corrected on cluster level, initial voxel threshold .001 uncorrected).

*Gender differences*

To test whether exercise effects were modulated by gender, we performed two-sample t-tests comparing exercise-related changes in behavioral performance and brain activation between males and females for the moderate and high-intensity exercise groups separately.

For *hit* trials of the Go/No-go task, males (n=16) in the moderate-intensity group showed greater exercise-related increases in brain activation than females (n=16) in one cluster with peak activation in the right precentral gyrus, extending to the supramarginal and middle frontal gyri (Table S1). For hit trials in the high-intensity group, we found no differences in exercise-related brain activation changes between males and females. There were neither gender-related differences in exercise-induced brain activation changes during the flanker or visual task nor differences in behavioral performance changes due to exercise.

Based on those results, we tested again for differences in condition-specific activation changes (exercise – control) during *hits* between the moderate and the high-intensity group including only male participants (moderate: n=16; high-intensity: n=15). We found significant differences in six clusters (Table S1): the first cluster was located in the left insula and extended to the orbital part of the inferior frontal gyrus and the middle frontal gyrus; the second cluster presented with a peak activation in the left inferior frontal operculum and included the left insula, rolandic operculum, and superior temporal pole; the third cluster comprised the right rolandic operculum, postcentral gyrus, inferior frontal operculum, and superior temporal pole; the fourth cluster extended from the right superior frontal gyrus to the midcingulate cortex, supplementary motor area, left and right precentral gyri, left middle frontal gyrus, and the right postcentral gyrus; the fifth cluster comprised the right rolandic operculum, insula, postcentral gyrus, superior temporal gyrus, and putamen; the sixth cluster extended from the right postcentral gyrus to right inferior and superior parietal regions and the precuneus. BOLD responses within those clusters increased in the moderate-intensity group and decreased in the high-intensity group in the exercise compared to the control condition. Peak coordinates and statistical results are given in Table S1.

**Table S1**

Gender differences in exercise-related brain activation changes during hit trials of the Go/No-go task

| **Group, Condition** | **Region of peak activation** | **MNI coordinates (x, y, z)** | **Cluster Size** | **t-statistic** | **z-statistic** | **p*** |
| --- | --- | --- | --- | --- | --- | --- |
| MI (exercise – control) |  |  |  |  |  |  |
| male – female^1^ | R precentral | 58, 4, 30 | 349 | 5.02 | 4.24 | .010 |
| female – male^1^ | not significant |  |  |  |  |  |
|  |  |  |  |  |  |  |
| HI (exercise – control) |  |  |  |  |  |  |
| male – female^1^ | not significant |  |  |  |  |  |
| female – male^1^ | not significant |  |  |  |  |  |
|  |  |  |  |  |  |  |
| MI (exercise – control) – HI (exercise – control)^1^ |  |  |  |  |  |  |
| only males | L insula | -34, 26, 8 | 530 | 6.28 | 4.95 | .001 |
|  | L inferior frontal operculum | -60, 8, 14 | 637 | 5.83 | 4.70 | <.001 |
|  | R rolandic operculum | 60, 2, 10 | 634 | 5.58 | 4.56 | <.001 |
|  | R superior frontal | 20, -12, 64 | 3423 | 5.42 | 4.47 | <.001 |
|  | R rolandic operculum | 42, -20, 18 | 353 | 5.05 | 4.24 | .008 |
|  | R postcentral | 30, -40, 54 | 303 | 4.40 | 3.82 | .015 |
|  |  |  |  |  |  |  |
| only females | not significant |  |  |  |  |  |

*FWE-corrected on cluster level (initial voxel threshold .001 uncorrected); ^1^ two-sample t-test.

MI = Moderate intensity; HI = High intensity.
